# Mental Health Outcomes and Associations During the COVID-19 Pandemic: A Cross-Sectional Population-Based Study in the United States

**DOI:** 10.3389/fpsyt.2020.569083

**Published:** 2020-12-09

**Authors:** Bella Nichole Kantor, Jonathan Kantor

**Affiliations:** ^1^Harvard Extension School, Harvard University, Cambridge, MA, United States; ^2^Center for Behavioral Epidemiology, St. Augustine, FL, United States; ^3^Center for Global Health, University of Pennsylvania Perelman School of Medicine, Philadelphia, PA, United States; ^4^Center for Clinical Epidemiology and Biostatistics, University of Pennsylvania Perelman School of Medicine, Philadelphia, PA, United States; ^5^Department of Dermatology, University of Pennsylvania Perelman School of Medicine, Philadelphia, PA, United States; ^6^Florida Center for Dermatology, St. Augustine, FL, United States

**Keywords:** COVID-19, mental health, pandemic (COVID-19), anxiety, depression

## Abstract

Pandemic coronavirus disease 2019 (COVID-19) may lead to significant mental health stresses, potentially with modifiable risk factors. We performed an internet-based cross-sectional survey of an age-, sex-, and race-stratified representative sample from the US general population. Degrees of anxiety, depression, and loneliness were assessed using the 7-item Generalized Anxiety Disorder-7 scale (GAD-7), the 9-item Patient Health Questionnaire, and the 8-item UCLA Loneliness Scale, respectively. Unadjusted and multivariable logistic regression analyses were performed to determine associations with baseline demographic characteristics. A total of 1,005 finished surveys were returned of the 1,020 started, yielding a completion rate of 98.5% in the survey panel. The mean (standard deviation) age of the respondents was 45 (16) years, and 494 (48.8%) were male. Overall, 264 subjects (26.8%) met the criteria for an anxiety disorder based on a GAD-7 cutoff of 10; a cutoff of 7 yielded 416 subjects (41.4%), meeting the clinical criteria for anxiety. On multivariable analysis, male sex (odds ratio [OR] = 0.65, 95% confidence interval [CI] [0.49, 0.87]), identification as Black (OR = 0.49, 95% CI [0.31, 0.77]), and living in a larger home (OR = 0.46, 95% CI [0.24, 0.88]) were associated with a decreased odds of meeting the anxiety criteria. Rural location (OR 1.39, 95% CI [1.03, 1.89]), loneliness (OR 4.92, 95% CI [3.18, 7.62]), and history of hospitalization (OR = 2.04, 95% CI [1.38, 3.03]) were associated with increased odds of meeting the anxiety criteria. Two hundred thirty-two subjects (23.6%) met the criteria for clinical depression. On multivariable analysis, male sex (OR = 0.71, 95% CI [0.53, 0.95]), identifying as Black (OR = 0.62, 95% CI [0.40, 0.97]), increased time outdoors (OR = 0.51, 95% CI [0.29, 0.92]), and living in a larger home (OR = 0.35, 95% CI [0.18, 0.69]) were associated with decreased odds of meeting depression criteria. Having lost a job (OR = 1.64, 95% CI [1.05, 2.54]), loneliness (OR = 10.42, 95% CI [6.26, 17.36]), and history of hospitalization (OR = 2.42, 95% CI [1.62, 3.62]) were associated with an increased odds of meeting depression criteria. Income, media consumption, and religiosity were not associated with mental health outcomes. Anxiety and depression are common in the US general population in the context of the COVID-19 pandemic and are associated with potentially modifiable factors.

## Introduction

The coronavirus disease 2019 (COVID-19) pandemic has led to unprecedented levels of movement restriction, job losses, and economic uncertainty in the United States and around the world (1). Concerns regarding illness, death, and the death of loved ones may be compounded by financial uncertainty, as reports of mass unemployment with variable international governmental responses circulate (2).

Mental health outcomes have been associated with pandemics in the past (3–5). While there has been a rapid response to the COVID-19 pandemic in terms of nonpharmaceutical interventions, vaccine development, and medical support, little comprehensive planning has been performed to predict and respond to the possible mental health crisis that could emerge from the pandemic, and the only data available on general public responses to the pandemic are in Chinese populations (6, 7). A recent study in the UK population included a set of two surveys of the general public, although their focus was on prioritization of concerns rather than estimating the prevalence of mental health outcomes (8). These data are echoed by research suggesting that healthcare workers have a significant burden of mental health challenges in the face of COVID-19 and highlighting the potential psychological effects of quarantine (9, 10). Moreover, pandemics and other natural disasters may disproportionately affect those with underlying mental illness, the elderly, and healthcare workers (11–14). Loneliness can be exacerbated by the COVID-19 pandemic through several mechanisms and may be associated with other mental health outcomes (15, 16). Time spent outdoors may also be associated with better mental health in the pandemic context, particularly if this time is spent exercising, a benefit that may be more pronounced for women than men (17). Time spent outdoors in general may also be associated with improved positive affect and decreased negative emotions based on a UK study (18). A recent position paper issued a call to action for high-quality population-level data to assess the mental health burden in the context of the present pandemic (8), and it is possible that the uptake of nonpharmaceutical interventions is affected by mental health outcomes given the role of behavioral considerations in their implementation (19, 20).

We therefore sought to investigate the prevalence of anxiety and depression in the general US population in the context of the early COVID-19 pandemic and explore associations of these mental health outcomes with loneliness (of particular concern given enhanced social distancing and isolation), health status, socioeconomic status, residence size, time spent outdoors, and other baseline demographic characteristics. These baseline characteristics were chosen given their potential relevance for developing future risk models and their potential modifiability. A better understanding of the prevalence of these mental health outcomes and their putative risk factors may help guide public policy, research, and interventions.

## Methods

### Study Design

This study is a cross-sectional, internet-based survey performed via age, sex, and race stratification to reflect the makeup of the general US population, conducted between March 29, 2020, and March 31, 2020. Responses to all survey questions were recorded (Supplemental file). This study was deemed exempt by the Ascension Health institutional review board.

We developed an online survey using the Qualtrics platform (Qualtrics Corp., Provo, UT) after iterative online pilot testing. The survey was distributed to a representative sample of the US population using Prolific Academic (Oxford, United Kingdom), an established platform for academic survey research (21). Prolific Academic maintains a database of possible survey respondents and distributes surveys using a survey panel approach. Respondents were rewarded with a small payment (<US $1). Participants provided consent and were permitted to terminate the survey at any time. All surveys were anonymous and confidential, with linkages between data performed using a 24-character alphanumeric code. The investigators had no access to identifying information at any time.

### Participants

This internet-based survey was stratified by age, sex, and race to reflect the makeup of the general US population. Sample size calculations were conducted for the primary endpoint of detecting a 10% difference in the Generalized Anxiety Disorder-7 scale (GAD-7) between those that were and were not under a stay-at-home order at the time of survey completion. Six hundred eighty-two subjects (341 per group) would be adequate to detect a 10% change in GAD-7 with 80% power and with an α of 0.05, assuming a baseline GAD-7 mean of 11.6 with a standard deviation (SD) of 5.4 and assuming equal group sizes (22). We inflated our sample size to 1,000, given that approximately two-thirds of the United States was under stay-at-home orders at the time of survey initiation and given uncertainty regarding changes in those orders over the duration of the survey, as well as to permit subgroup analyses.

### Outcome Measures and Independent Variables

For our main outcome measures, anxiety and depression, validated scales were used. Anxiety was assessed using the GAD-7, a validated self-report scale for anxiety, with scores ranging from 0 (no anxiety) to 21 (extreme anxiety). Prior psychometric research suggested cutoffs as 0–4 (no anxiety), 5–9 (mild anxiety), 10–14 (moderate anxiety), and 15–21 (severe anxiety) (9, 22).

Depression was assessed with the Patient Health Questionnaire-9 (PHQ-9), a validated measure for clinical depression (23). Scores range from 0 (no depression) to 27 (severe depression). Prior psychometric research has suggested cutoffs as 0–4 (no depression), 5–9 (mild depression), 10–14 (moderate depression), and 15–27 (severe depression) (9, 24).

Loneliness was quantified with the UCLA Short-Form Loneliness Scale (ULS-8), a validated measure of loneliness (25). Scores range from 8 (no loneliness) to 32 (extreme loneliness); no clinically meaningful cutoffs have been established psychometrically.

Age, sex, rural area residence, and race were included based on self-report. Binary choices were provided for sex selection. Race was divided into six categories: White, Black/African American, American Indian/Alaskan Native, Asian, Native Hawaiian/Pacific Islander, and other. Religiosity was defined based on self-report of whether the participant considered themselves a religious person, including an ambivalent option. Media consumption was assessed by the number of hours spent watching or reading about the pandemic over the preceding 3 days. Time spent outdoors was measured by self-report on number of hours spent outside over the preceding 3 days, with options for 0, <1, 1–3, 3–5, and more than 5. Income was based on self-report using a range of 12 options from <$10,000 per year to more than $150,000 per year. Home size was based on self-report, with options ranging from <500 square feet to more than 2,000 square feet.

### Statistical Analysis

Normally distributed baseline demographic data are presented as mean values with 95% confidence intervals (CIs). Outcomes that were not normally distributed are presented as medians with interquartile ranges (IQRs). *t* Tests and χ^2^ tests were used as appropriate for baseline continuous and categorical variables. Subgroup comparisons of non–normally distributed data were performed using the Kruskal–Wallis test. Unadjusted and multivariable (adjusting for the nonmodifiable variables of age and sex) logistic regression odds ratios (ORs) of association were assessed between the dependent variables of anxiety or depression, presented as dichotomous outcomes using the established cutoffs of 10 for both the GAD-7 and PHQ-9, and putative risk factors.

All statistical analyses were performed using Stata 13 for Mac (Stata Corp., College Station, TX).

## Results

### Baseline Characteristics

Of the 1,020 subjects who were recruited, 1,005 finished the survey, yielding a completion rate of 98.5%. The mean (SD) age of the respondents was 45 (16) years, and 494 (48.8%) of the respondents were male; baseline respondent characteristics are outlined in Table 1. Baseline demographic data were similar between those that were (*n* = 663, 66.2%) and were not (*n* = 339, 33.8%) under a shelter-in-place or stay-at-home order (defined by self-report), with the exception of sex and geographic location (urban vs. rural status). The median (IQR) ULS-8 score for loneliness was 16 (12–20), similar to baseline estimates from previous studies (25–27).

**Table 1:** Demographic and baseline characteristics of respondents, overall, and by shelter-in-place status.

**Characteristic**	**No. (%)**		
		**Under a shelter-in-place order**	
	**Total**	**Yes**	**No**
Overall	1,005 (100)	681 (66. 8)	389 (33.2)
Sex^*^			
Men	494 (48.8)	310 (46.1)	184 (54.3)
Women	518 (51.2)	363 (53.9)	155 (45.7)
**Age, y**			
18–30	250 (24.5)	165 (24.2)	85 (25.1)
31–40	204 (20.0)	139 (20.4)	65 (19.2)
41–50	146 (14.3)	100 (14.7)	46 (13.6)
51–60	198 (198.4)	130 (19.1)	68 (20.1)
>60	222 (21.8)	147 (21.6)	75 (22.1)
**Race**			
Black	138 (13.8)	94 (14.2)	33 (13.0)
White	761 (76.0)	490 (74.2)	268 (79.1)
American Indian	6 (0.60)	3 (0.5)	3 (0.9)
Asian	62 (6.2)	47 (7.1)	15 (4.4)
Pacific Islander	4 (0.40)	4 (0.6)	0 (0)
Other	31 (3.1)	22 (3.3)	9 (2.7)
**Education level**			
<High school	11 (1.1)	8 (1.2)	3 (0.9)
High school	117 (11.7)	67 (10.1)	50 (14.8)
Some college	228 (22.8)	149 (22.4)	79 (23.4)
Associates	103 (10.3)	66 (9.9)	37 (11.0)
Bachelor's	358 (35.7)	246 (37.0)	112 (33.2)
Graduate	185 (18.5)	129 (19.4)	56 (16.6)
**Employment status**			
Full time	461 (45.2)	303 (44.5)	158 (46.6)
Part time	170 (16.7)	115 (16.9)	55 (16.2)
Not employed	389 (38.1)	263 (38.6)	127 (37.2)
**Marital status**			
Married	414 (40.6)	273 (41.2)	139 (41.0)
Unmarried	606 (59.4)	390 (58.8)	200 (59.0)
**Religious**			
Yes	387 (37.9)	252 (37.0)	135 (39.8)
No	543 (53.2)	361 (53.0)	182 (53.7)
Ambivalent	90 (8.8)	68 (10.0)	22 (6.5)
**Income**			
<$10,000	167 (16.4)	115 (16.9)	52 (15.3)
$10,000–$30,000	234 (22.9)	154 (22.6)	80 (23.6)
$30,001–$50,000	220 (21.6)	137 (20.1)	83 (24.5)
$50,001–$80,000	201 (19.7)	131 (19.2)	70 (20.7)
$80,001–$100,000	63 (6.2)	42 (6.2)	21 (6.2)
$100,001–$150,000	91 (8.9)	71 (10.4)	20 (5.9)
>$150,000	44 (4.3)	31 (4.6)	13 (3.8)
Location^*^			
Urban	743 (72.8)	517 (75.9)	226 (66.7)
Rural	277 (27.2)	164 (24.1)	113 (33.3)

* *p < 0.05*.

### Anxiety

The median (IQR) GAD-7 score was 5 (1–10), and 513 subjects (52.1%) of the subjects had at least mild anxiety. Median GAD-7 scores did not differ significantly by shelter-in-place order status (*p* = 0.128). Overall, 264 subjects (26.8%) met the criteria for an anxiety disorder based on a GAD-7 cutoff of 10 (Table 2). Adopting a more liberal GAD-7 cutoff of 7, as used in a recent study on healthcare worker anxiety in the COVID-19 context (9), would yield 416 subjects (41.4%) meeting the clinical criteria for anxiety. Women (*p* = 0.002) and those living in rural areas (*p* = 0.041) reported more severe anxiety than men and those in urban areas, respectively.

**Table 2:** Anxiety and depression severity, by selected baseline characteristics.

**Severity**	**No (%)**												
		**Sex**			**Shelter-in-place**			**Location**			**Married**		
	**Overall**	**Male**	**Female**		**Yes**	**No**		**Urban**	**Rural**		**Yes**	**No**	
**GAD-7**													
None	472 (47.9)	260 (53.9)	212 (42.2)	*P* = 0.002	299 (45.9)	172 (52.1)	*P* = 0.314	337 (47.5)	134 (49.5)	*P* = 0.041	195 (48.2)	277 (47.8)	*P* = 0.894
Mild	249 (25.3)	111 (23.0)	138 (27.4)		172 (26.4)	76 (23.0)		194 (27.4)	53 (19.6)		106 (27.2)	143 (24.7)	
Moderate	132 (13.4)	60 (12.5)	72 (14.3)		89 (13.7)	42 (12.7)		93 (13.1)	39 (14.4)		51 (12.6)	81 (14.0)	
Severe	132 (13.4)	51 (10.6)	81 (16.1)		92 (14.1)	40 (12.1)		85 (12.0)	45 (16.6)		53 (13.1)	79 (13.6)	
**PHQ-9**													
None	518 (52.7)	278 (58.3)	240 (47.4)	*P* = 0.008	340 (52.2)	176 (53.3)	*P* = 0.457	373 (52.8)	143 (52.6)	*P* = 0.714	239 (59.9)	279 (47.8)	*P* < 0.0001
Mild	233 (23.7)	102 (21.4)	131 (25.9)		149 (22.9)	84 (25.5)		165 (23.4)	68 (25.0)		92 (23.10	141 (24.1)	
Moderate	116 (11.8)	48 (10.1)	68 (13.4)		84 (12.9)	32 (9.7)		114 (11.7)	27 (9.9)		34 (8.5)	82 (14.0)	
Severe	116 (11.8)	49 (10.3)	67 (13.2)		78 (12.0)	38 (11.5)		115 (11.8)	34 (12.5)		34 (8.5)	82 (14.0)	

*GAD-7, Generalized Anxiety Disorders scale; PHQ-9, Patient Health Questionnaire*.

Unadjusted logistic regression analysis demonstrated that men (OR = 0.67, 95% CI [0.51, 0.89]) and those who identified as Black (OR = 0.63, 95% CI [0.40, 0.97]) were less likely to meet the criteria for anxiety, whereas, those who lost their job (OR = 1.61, 95% CI [1.45, 2.45]), had been hospitalized within the past 2 years (OR = 1.86, 95% CI [1.27, 2.73]), or were in the most lonely quartile (OR = 5.39, 95% CI [3.53, 8.24]) were more likely to meet the criteria for anxiety. On multivariable analysis controlling for age and sex as confounders, male sex (OR = 0.65, 95% CI [0.49, 0.87]), identifying as Black (OR = 0.49, 95% CI [0.31, 0.77]), and living in a larger home (OR = 0.46, 95% CI [0.24, 0.88]) were associated with a decreased odds of meeting the anxiety criteria. Rural location (OR = 1.39, 95% CI [1.03, 1.89]), loneliness (OR = 4.92, 95% CI [3.18, 7.62]), and history of hospitalization within the past 2 years (OR = 2.04, 95% CI [1.38, 3.03]) were independent risk factors for meeting the anxiety criteria (Table 3). In a fully adjusted model including all variables that demonstrated *p* > 0.10 on univariable analyses, identifying as Asian (OR = 0.28, 95% CI [0.10, 0.79]), male sex (OR = 0.59, 95% CI [0.38, 0.94]), and having a larger home size (OR = 0.14, 95% CI [0.04, 0.42] for homes larger than 2,000 square feet) were associated with a decreased odds of meeting the anxiety criteria. Loneliness (OR = 5.05, 95% CI [2.90, 8.78]) was associated with an increased odds of meeting the anxiety criteria in the fully adjusted model.

**Table 3:** Risk factors for anxiety and depression in unadjusted and multivariable analyses.

	**Unadjusted OR (95% CI)**	***P* value**	**Adjusted^†^ OR (95% CI)**	***P* value**
GAD-7 (Anxiety)^*^				
**Sex**				
Male	0.67 (0.51, 0.89)	0.005	0.65 (0.49, 0.87)	0.003
Female	1 [Reference]		1 [Reference]	
**Location**				
Rural	1.32 (0.98, 1.79)	0.066	1.39 (1.03, 1.89)	0.034
Urban	1 [Reference]		1 [Reference]	
**Religious**				
No	1 [Reference]		1 [Reference]	
Yes	0.85 (0.63, 1.14)	0.267		
Ambivalent	1.21 (0.75, 1.96)	0.425		
**Hospitalized in past 2 years**				
Yes	1.86 (1.27, 2.73)	0.001	2.04 (1.38, 3.03)	<0.0001
No	1 [Reference]		1 [Reference]	
**Lost job due to COVID-19**				
Yes	1.61 (1.05, 2.45)	0.028	1.53 (0.99, 2.35)	0.055
No	1 [Reference]		1 [Reference]	
**Loneliness (ULS-8)**^**‡**^				
Most lonely quartile	5.39 (3.53, 8.24)	<0.0001	4.92 (3.18, 7.62)	<0.0001
Least lonely quartile	1 [Reference]		1 [Reference]	
**Home size (square feet)**				
<500	1 [Reference]		1 [Reference]	
500–750	0.59 (0.32, 1.12)	0.107	0.60 (0.31, 1.14)	0.120
750–1,000	0.38 (0.20, 0.70)	0.002	0.37 (0.20, 0.69)	0.002
1,000–1,500	0.48 (0.27, 0.85)	0.013	0.50 (0.28, 0.91)	0.024
1,500–2,000	0.50 (0.27, 0.90)	0.022	0.53 (0.29, 0.98)	0.043
>2,000	0.39 (0.21, 0.74)	0.004	0.46 (0.24, 0.88)	0.019
**Time spent outdoors in past 3 days (hours)**				
0	1 [Reference]		1 [Reference]	
<1	0.78 (0.47, 1.30)	0.339	0.79 (0.47, 1.32)	0.366
1–3	0.74 (0.46, 1.20)	0.226	0.77 (0.47, 1.27)	0.303
3–5	0.62 (0.34, 1.12)	0.115	0.72 (0.39, 1.31)	0.278
>5	0.83 (0.48, 1.43)	0.504	0.95 (0.54, 1.65)	0.849
**Income**				
<$10,000	1 [Reference]		1 [Reference]	
$10,000–$30,000	0.61 (0.40, 0.93)	0.021	0.78 (0.50, 1.21)	0.259
$30,001–$50,000	0.60 (0.39, 0.93)	0.022	0.79 (0.50, 1.23)	0.292
$50,001–$80,000	0.52 (0.33, 0.81)	0.004	0.69 (0.44, 1.11)	0.125
$80,001–$100,000	0.46 (0.24, 0.90)	0.023	0.61, 0.31, 1.22)	0.161
$100,001–$150,000	0.54 (0.29, 0.98)	0.044	0.72 (0.39, 1.34)	0.296
>$150,000	0.75 (0.37, 1.52)	0.427	1.08 (0.52, 2.24)	0.832
**Education level**				
<High school	1 [Reference]		1 [Reference]	
High school	0.88 (0.24, 3.17)	0.839	1.15 (0.31, 4.28)	0.831
Some college	0.73 (0.21, 2.57)	0.622	0.94 (0.26 3.42)	0.931
Associates	0.56 (0.15, 2.08)	0.386	0.74 (0.19, 2.81)	0.657
Bachelor's	0.66 (0.19, 2.30)	0.514	0.90 (0.25, 3.21)	0.866
Graduate	0.67 (0.19, 2.37)	0.531	1.00 (0.27, 3.65)	0.996
**Race**				
White	1 [Reference]		1 [Reference]	
Black	0.63 (0.40, 0.97)	0.036	0.49 (0.31, 0.77)	0.002
American Indian	1.18 (0.21, 6.47)	0.852	0.70 (0.12, 3.96)	0.683
Asian	0.69 (0.37, 1.27)	0.230	0.51 (0.27, 0.95)	0.035
Pacific Islander	7.06 (0.73, 68.21)	0.091	4.95 (0.50, 48.92)	0.171
Other	0.69 (0.29, 1.62)	0.388	0.57 (0.24, 1.37)	0.208
PHQ-9 (Depression)^*^				
**Sex**				
Male	0.73 (0.55, 0.98)	0.034	0.71 (0.53, 0.95	0.023
Female	1 [Reference]		1 [Reference]	
**Location**				
Rural	0.90 (0.65, 1.24)	0.515	0.94 (0.67, 1.30)	0.700
Urban	1 [Reference]		1 [Reference]	
**Hospitalized in past 2 years**				
Yes	2.16 (1.47, 3.17)	<0.0001	2.42 (1.62, 3.62)	<0.0001
No	1 [Reference]		1 [Reference]	
**Lost job due to COVID-19**				
Yes	1.74 (1.13, 2.67)	0.011	1.64 (1.05, 2.54)	0.029
No	1 [Reference]		1 [Reference]	
**Loneliness (ULS-8)**^**‡**^				
Most lonely quartile	11.90 (7.21, 19.65)	<0.0001	10.42 (6.26, 17.36)	<0.0001
Least lonely quartile	1 [Reference]		1 [Reference]	
**Home size (square feet)**				
<500	1 [Reference]		1 [Reference]	
500–750	0.50 (0.26, 0.94)	0.033	0.50 (0.26, 0.96)	0.037
750–10,000	0.41 (0.22, 0.76)	0.004	0.40 (0.22, 0.76)	0.005
1,000–1,500	0.42 (0.23, 0.76)	0.004	0.45 (0.25, 0.83)	0.010
1,500–2,000	0.35 (0.19, 0.65)	0.001	0.38 (0.20, 0.72)	0.003
>2,000	0.29 (0.15, 0.56)	<0.0001	0.35 (0.18, 0.69)	0.002
**Time spent outdoors in past 3 days (hours)**				
0	1 [Reference]		1 [Reference]	
<1	0.77 (0.47, 1.27)	0.302	0.80 (0.48, 1.34)	0.398
1–3	0.55 (0.34, 0.88)	0.014	0.58 (0.35, 0.95)	0.031
3–5	0.45 (0.24, 0.82)	0.010	0.53 (0.28, 0.98)	0.044
>5	0.45 (0.25, 0.79)	0.006	0.51 (0.29, 0.92)	0.025
**Income**				
<$10,000	1 [Reference]		1 [Reference]	
$10,000–$30,000	0.58 (0.38, 0.89)	0.012	0.78 (0.50, 1.21)	0.264
$30,001–$50,000	0.45 (0.29, 0.70)	<0.0001	0.59 (0.37, 0.94)	0.026
$50,001–$80,000	0.46 (0.29, 0.73)	0.001	0.63 (0.39, 1.02)	0.060
$80,001–$100,000	0.38 (0.19, 0.76)	0.007	0.51 (0.25, 1.04)	0.064
$100,001–$150,000	0.40 (0.21, 0.75)	0.005	0.53 (0.27, 1.02)	0.059
>$150,000	0.36 (0.16, 0.82)	0.015	0.52 (0.22, 1.20)	0.126
**Education level**				
<High school	1 [Reference]		1 [Reference]	
High school	1.23 (0.31, 4.92)	0.766	1.76 (0.43, 7.19)	0.432
Some college	0.97 (0.25, 3.79)	0.970	1.39 (0.35, 5.54)	0.642
Associates	0.68 (0.17, 2.80)	0.596	0.99 (0.24, 4.17)	0.992
Bachelor's	0.80 (0.21, 3.10)	0.753	1.19 (0.30, 4.71)	0.801
Graduate	0.91 (0.23, 3.57)	0.890	1.53 (0.38, 6.17)	0.553
**Race**				
White	1 [Reference]		1 [Reference]	
Black	0.81 (0.52, 1.25)	0.333	0.62 (0.40, 0.97)	0.038
Asian	0.93 (0.51, 1.69)	0.804	0.66 (0.36, 1.23)	0.194
Pacific Islander	8.71 (0.90, 84.20)	0.062	6.10 (0.61, 60.87)	0.123
Other	1.19 (0.54, 2.62)	0.671	0.99 (0.44, 2.25)	0.983

* *Score cutoff of 10 used for classification*.

† *Adjusted for age and sex*.

‡ *Assessed using the UCLA Loneliness Scale*.

*GAD-7, Generalized Anxiety Disorders scale; PHQ-9, Patient Health Questionnaire*.

### Depression

The median (IQR) PHQ-9 score was 4 (1–9), and 465 (47.3%) of the subjects reported at least mild depression by screening (Table 2). Median PHQ-9 scores did not differ significantly by shelter-in-place order status (*p* = 0.743). A total of 232 subjects (23.6%) met the criteria for clinical depression. Women (*p* = 0.008) and unmarried subjects (*p* < 0.0001) reported more severe depression than men and those who are married, respectively.

Unadjusted logistic regression analysis demonstrated that men were less likely to meet the criteria for depression (OR = 0.73, 95% CI [0.55, 0.98]), whereas those who lost their job (OR = 1.74, 95% CI [1.13, 2.67]), had been hospitalized within the past 2 years (OR = 2.16, 95% CI [1.47, 3.17]), or were in the most lonely quartile (OR = 11.90, 95% CI [7.21, 19.65]) were more likely to meet the criteria for depression. On multivariable analysis controlling for age and sex as confounders, male sex (OR = 0.71, 95% CI [0.53, 0.95]), identifying as Black (OR = 0.62, 95% CI [0.40, 0.97]), increased time outdoors (OR = 0.51, 95% CI [0.29, 0.92]), and living in a larger home (OR = 0.35, 95% CI [0.18, 0.69]) were associated with a decreased odds of meeting depression criteria. Having lost a job (OR = 1.64, 95% CI [1.05, 2.54]), loneliness (OR = 10.42, 95% CI [6.26, 17.36]), and history of hospitalization within the past 2 years (OR = 2.42, 95% CI [1.62, 3.62]) were associated with meeting depression criteria (Table 3). In a fully adjusted model including all variables that demonstrated *p* > 0.10 on univariable analyses, identifying as Black (OR = 0.47, 95% CI [0.23, 0.98]), identifying as Asian (OR = 0.32, 95% CI [0.12, 0.85]), increasing age (OR = 0.98, 95% CI [0.96, 1.00]), and spending 5 h or more outside over the past 3 days (OR = 0.36, 95% CI [0.14, 0.91]) were associated with a decreased odds of meeting depression criteria. Loneliness (OR = 9.41, 95% CI [4.99, 17.75]) was associated with an increased odds of meeting depression criteria in the fully adjusted model.

## Discussion

In this first study of general US population mental health during the COVID-19 pandemic, we found high baseline levels of both anxiety and depression, independent of living under a shelter-in-place or stay-at-home order. More than half (52.1%) of the respondents had at least mild anxiety, and 47.3% of the subjects had at least mild depressive symptoms. This high burden of mental health concerns in the general population in the pandemic context suggests the need for further study and consideration for intervention. That said, the prevalence of mental health outcomes is fairly high in the US population at baseline, where estimates have suggested the prevalence of anxiety and depression is on the order of 19% and 24%, respectively (28, 29).

We found that respondents who identify as Black were less likely to meet the criteria for anxiety on both univariate and adjusted analyses and less likely to meet the criteria for depression on adjusted analyses. Those who identified as Asian had decreased odds of meeting both anxiety and depression on fully adjusted analyses. This finding suggests the need for further study, although they echo prepandemic research that has suggested a lower prevalence of self-reported anxiety in respondents who identify as Black (30–32). Estimates regarding the prevalence of depression vary, with some studies suggesting a higher prevalence among minorities and others pointing to a lower prevalence among racial minorities (33–36). Further research is needed to determine whether this finding is a result of confounding or chance or is replicable.

Living in a larger home was associated with a reduced risk of both anxiety and depression; this effect was seen despite the lack of any association between anxiety or depression and household income and persisted when including income and number of household members in a multivariable model. Similarly, we found that increased time spent outdoors correlated with a reduction in depression (but not anxiety) risk, and those who spent more than an hour a day outdoors had approximately half the risk of depression as those who spent no time outdoors. This association of depression with time outdoors echoes prior research on associations with time spent outdoors and its impact on mental health (37, 38). Our finding that both larger living space and increased time spent outdoors correlate with a reduction in mental health burden may have actionable implications for public health initiatives and decisions regarding access to outdoor recreation areas during stay-at-home or shelter-in-place orders.

History of hospitalization, a rough measure of overall health status, was associated with an increased risk of both anxiety and depression. This effect persisted even when controlling for age and history of anxiety and depression, respectively, suggesting that those with a poorer health status may be at increased risk of adverse mental health outcomes in the context of the COVID-19 pandemic.

Media consumption was not associated with the presence of anxiety or depression. Similarly, we did not detect significant associations between likelihood of meeting the criteria for anxiety or depression and household income or religiosity on adjusted multivariable analyses.

Notably, we found that fewer than half of the respondents had no anxiety; that is, more than half of the subjects reported a level of anxiety that would at least be classified as mild. Conversely, 13.4% of the subjects demonstrated severe anxiety, a higher proportion than has been reported even in healthcare workers responding to pandemic COVID-19 (9). Our finding that 23.6% of the subjects met the criteria for depression using the PHQ-9 echoes earlier pooled data that suggested a prevalence of 24.6% for depression using this scale, although this pooled estimate from 44 studies may overestimate the baseline prevalence of depression due to the inherent limitations of the PHQ-9 (29). The heterogeneity of baseline measures of depression as measured by the PHQ-9 has also been reported in assessments of outpatients (39).

Loneliness is an established risk factor for both anxiety and depression (26, 40), and we found an ~5- to 10-fold increase in odds of anxiety and depression, respectively, with being in the highest loneliness quartile. That said, there may be a tautological relationship between loneliness and depression, and this should be considered when evaluating the relationship between these variables (41). As with those living in smaller homes with minimal access to the outdoors, loneliness can be seen as an independent risk factor for anxiety or depression in the context of the COVID-19 pandemic.

This study has several limitations. First, as with any survey-based research, its generalizability may be limited. We used Prolific Academic for survey distribution in order to maximize our generalizability to the general US population by using an age-, sex-, and race-stratified survey panel design with a large and validated sampling frame. As with any survey data, however, the sample willing to participate may not fully reflect the population of interest, and stratifying by these variables does not guarantee that the sample population reflects the general population in any other way. Second, our study took place during the early phase of the COVID-19 pandemic, when shelter-in-place and stay-at-home orders were only just beginning. If anything, however, this underestimates the prevalence of anxiety and depression as these outcomes would only be expected to increase as restrictions persist and highlights that even the anticipation of such restrictions may present a stressor. Third, as with any survey study, response bias and social desirability bias may play a role, although the anonymous survey design may help mitigate these concerns. Fourth, while our study relied on validated scales wherever possible, some survey questions were the product of pilot testing alone, and therefore their methodology—although consistent with the survey development literature—has not been fully vetted. Fifth, our selection of independent variables was not exhaustive, and other important variables, such as sleep (42), family stress (43), underlying mental health diseases (44), and others, may be important confounders. Finally, and importantly, this cross-sectional study that lacks a comparator group cannot establish causation; therefore, we do not know whether the associations we describe are truly clinical risk factors.

In this first study of mental health outcomes in the US population during the COVID-19 pandemic, we found high rates of depression and anxiety, with the most profound mental health effects in women, those with a history of hospitalization over the past 2 years, those who were most lonely, and those living in smaller homes, and (for depression) those spending the least time outdoors. These findings may be considered in future public health efforts and in developing national and international pandemic responses.

## Data Availability Statement

The raw data supporting the conclusions of this article will be made available by the authors, without undue reservation.

## Ethics Statement

The studies involving human participants were reviewed and approved by Ascension Health IRB. The patients/participants provided their written informed consent to participate in this study.

## Author Contributions

BK and JK: study conception, design, writing, statistics. JK: oversight. All authors contributed to the article and approved the submitted version.

## Conflict of Interest

The authors declare that the research was conducted in the absence of any commercial or financial relationships that could be construed as a potential conflict of interest.
